# Reliability of Time-Series Plasma Metabolome Data over 6 Years in a Large-Scale Cohort Study

**DOI:** 10.3390/metabo14010077

**Published:** 2024-01-22

**Authors:** Atsuko Miyake, Sei Harada, Daisuke Sugiyama, Minako Matsumoto, Aya Hirata, Naoko Miyagawa, Ryota Toki, Shun Edagawa, Kazuyo Kuwabara, Tomonori Okamura, Asako Sato, Kaori Amano, Akiyoshi Hirayama, Masahiro Sugimoto, Tomoyoshi Soga, Masaru Tomita, Kazuharu Arakawa, Toru Takebayashi, Miho Iida

**Affiliations:** 1 Department of Preventive Medicine and Public Health, Keio University School of Medicine, Shinjuku, Tokyo 160-8582, Japan; atsuko.miyake@keio.jp (A.M.); seiharada@keio.jp (S.H.); dsugiyama@keio.jp (D.S.); minako@a7.keio.jp (M.M.); aya.hirata@keio.jp (A.H.); nmiya@keio.jp (N.M.); tk0523@keio.jp (R.T.); shun-edagawa@keio.jp (S.E.); kuwabara@keio.jp (K.K.); okamura@z6.keio.jp (T.O.); ttakebayashi@a3.keio.jp (T.T.); 2 Department of Obstetrics and Gynecology, Keio University School of Medicine, Shinjuku, Tokyo 160-8582, Japan; 3 Faculty of Nursing and Medical Care, Keio University, Kanagawa, Fujisawa 252-0883, Japan; 4 Graduate School of Health Management, Keio University, Kanagawa, Fujisawa 252-0883, Japan; 5 Institute for Advanced Biosciences, Keio University, Yamagata, Tsuruoka 997-0052, Japan; asako325@ttck.keio.ac.jp (A.S.); k-amano@ttck.keio.ac.jp (K.A.); hirayama@ttck.keio.ac.jp (A.H.); msugi@sfc.keio.ac.jp (M.S.); soga@sfc.keio.ac.jp (T.S.); mt@sfc.keio.ac.jp (M.T.); gaou@sfc.keio.ac.jp (K.A.)

**Keywords:** metabolomics, capillary electrophoresis–mass spectrometry, reliability, time-series data, repeated measurements, longitudinal data, cohort studies

## Abstract

Studies examining long-term longitudinal metabolomic data and their reliability in large-scale populations are limited. Therefore, we aimed to evaluate the reliability of repeated measurements of plasma metabolites in a prospective cohort setting and to explore intra-individual concentration changes at three time points over a 6-year period. The study participants included 2999 individuals (1317 men and 1682 women) from the Tsuruoka Metabolomics Cohort Study, who participated in all three surveys—at baseline, 3 years, and 6 years. In each survey, 94 plasma metabolites were quantified for each individual and quality control (QC) sample. The coefficients of variation of QC, intraclass correlation coefficients, and change rates of QC were calculated for each metabolite, and their reliability was classified into three categories: excellent, fair to good, and poor. Seventy-six percent (71/94) of metabolites were classified as fair to good or better. Of the 39 metabolites grouped as excellent, 29 (74%) in men and 26 (67%) in women showed significant intra-individual changes over 6 years. Overall, our study demonstrated a high degree of reliability for repeated metabolome measurements. Many highly reliable metabolites showed significant changes over the 6-year period, suggesting that repeated longitudinal metabolome measurements are useful for epidemiological studies.

## 1. Introduction

In recent years, large-scale metabolomic studies have been conducted worldwide. Large sample sizes, often in thousands, are particularly important to achieve sufficient reproducibility for biomarker discovery [1,2,3]. To date, a broad range of evidence has been obtained as a result of epidemiological studies using metabolomic data from single data points; however, the addition of metabolic profiling across multiple time points in a longitudinal setting will allow for a more precise estimation of the causal effects of metabolic variation [4,5]. Therefore, repeated longitudinal measurements of metabolomic data in cohorts spanning several years are expected to be more promising for future studies.

However, longitudinal studies can introduce new biases, such as changes in sample collection and storage as well as changes in biochemical assays caused by the long intervals between studies [6]. Statistically separating the analytical variation introduced by these experimental processes and instrumental analyses from the “true” biological variability is difficult [7]. To the best of our knowledge, few studies have examined the reliability of quantitative time-series metabolomic data in large populations [6,8].

Several methods are commonly used for metabolite analysis, among which capillary electrophoresis–mass spectrometry (CE-MS) has the advantages of higher separation efficiency and compound identification capability, allowing for the absolute quantification of polar metabolites, such as carbohydrates and amino acids [9,10], compared with other metabolome profiling methods.

The Tsuruoka Metabolomics Cohort Study (TMCS) is an ongoing prospective cohort study in Japan, for which plasma and urine metabolomic data was collected from more than 10,000 individuals across multiple time points using CE-MS methods. The TMCS aims to discover metabolomic biomarkers of common diseases and disorders related to genetic and environmental factors [11]. After the baseline study in 2012–2014 (Wave 1), follow-up studies were conducted 3 years later in 2015–2017 (Wave 2), 6 years later in 2018–2021 (Wave 3), and the third follow-up study (Wave 4) is underway as of 2023. We previously showed that large-scale metabolic profiling using CE-MS provided sufficiently high reproducibility and validity for both plasma and urine samples [10,11]. Liquid chromatography (LC)-MS was also incorporated in Wave 1 to expand metabolome coverage [12]; however, CE-MS has been mainly used for subsequent time-series measurements because of its high absolute quantitative accuracy. Therefore, CE-MS is considered suitable for assessing changes over time.

Considering the lack of studies examining the reliability of quantitative time-series metabolomic data in large populations, this study examined the reliability of repeatedly measuring plasma metabolites in a prospective cohort setting. We also explored the intra-individual changes in plasma metabolite concentrations over 6 years to discuss the utility of long-term repeated metabolome measurements in epidemiological studies.

## 2. Materials and Methods

### 2.1. Tsuruoka Metabolomics Cohort Study

In total, 11,002 participants aged 35–74 years (59.6 ± 10.1 years, 53% women) participated in Wave 1 from April 2012 to March 2015. All participants completed a comprehensive questionnaire on their lifestyle, dietary habits, and medical history. We also collected biological samples, including serum, plasma, urine, and buffy coats, as well as the data from health check-up programs provided by the municipality, employers, or employment-based insurers, including laboratory tests and physical examinations. Follow-up surveys were conducted and completed every 3 years, referred to as Waves 2 and 3; samples and questionnaire data were also collected during these later surveys. Information on death, onset of cardiovascular diseases and cancer, and other medical and laboratory data were also collected annually using national registry data, hospital records, and/or municipal or employment-based health checkups.

In Waves 2 and 3, conducted at 3 (3.0 ± 0.2) and 6 years (5.8 ± 0.8) after Wave 1, 4707 (56% women) and 6050 (55% women) participants were surveyed, respectively. The profile of the TMCS has been described in detail previously [11].

This study was approved by the Medical Ethics Committee of the Keio University School of Medicine, Tokyo, Japan (approval no. 20110264), and all participants provided written informed consent.

### 2.2. Study Subjects and Sample Collection

In total, 3314 individuals participated in all three surveys with a complete three-point metabolomic dataset. After excluding participants with a history of cerebral and cardiovascular diseases, such as myocardial infarction and stroke (121 subjects), or malignancy (161 subjects) at Wave 1, and 33 subjects for whom analyses were performed from a non-fasting blood draw, 2999 participants (1317 men (aged 55.1 ± 10.4 years) and 1682 women (aged 54.6 ± 10.6 years)) were included in this study.

Blood samples were collected after 12 h of overnight fasting to avoid dietary and circadian rhythm variations. Plasma samples were collected with EDTA-2Na as an anticoagulant and kept at 4 °C immediately after collection. Within 3 h after collection, the samples were centrifuged for 15 min (1500× *g* at 4 °C), divided into aliquots, and preserved at 4 °C until extraction of metabolites. To inhibit metabolic reactions in plasma, metabolite extraction from plasma was completed within 6 h after collection, the extract was then stored at −80 °C. Fifty microliters of plasma was used for sample extraction as previously described [13].

### 2.3. Metabolomics Measurements and Quality Control Samples

Metabolomic profiling of fasting plasma samples was conducted using capillary electrophoresis time-of-flight mass spectrometry (CE-TOF-MS). CE-TOF-MS analysis of cationic and anionic metabolites was performed as previously described [13,14]. Raw data were processed using our proprietary software (MasterHands ver.2) [13], and the absolute concentrations of 94 metabolites (54 cations and 40 anions), which were expected to be detected in more than 20% of the plasma samples based on our preliminary study, were measured [10].

We used three CE-MS instruments to measure cations and two to measure anions. These five instruments were used exclusively during the study period. Mass calibration using tuning solutions and MS entrance cleaning were performed at the beginning of every sequence to ensure a robust performance. Further, the number of samples per run was limited to 100 to avoid unexpected changes in the sensitivity of and variance in the measurement of MS in a continuous run.

**Figure 1:**
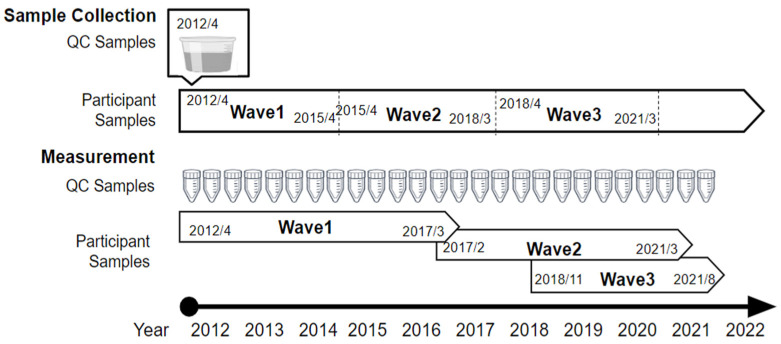
Sample collection and measurement process. QC samples were collected and pooled at the beginning of the study to be repeatedly measured for monitoring. Participant samples were collected during each study participation period (Waves 1, 2, and 3) and measured in sequential order. QC: quality control.

The metabolomic profiles of participant samples were analyzed in the order of collection beginning in April 2012, with 21,684 sample analyses completed by August 2021. These data comprised 238 running batches of cations and 231 batches of anions. The sample collection and metabolomics measurement processes are illustrated in Figure 1. The median measurement months were March 2016 for Wave 1, June 2017 for Wave 2, and July 2019 for Wave 3, for both cations and anions. To monitor the stability of the metabolomic analysis, quality control (QC) samples were injected for every 10 participant samples and assessed at the beginning of the analytical run and at intervals throughout the analysis. For QC samples, 150 mL serum collected in advance from 20 individuals from the same population was extracted for metabolomics analysis immediately after collection, then divided into 50 μL aliquots and stored at −80 °C. The QC aliquots were thawed and used for monitoring during the study. We calculated the mean concentration of each metabolite in the QC samples that were previously analyzed in 70 sequences. When the concentration of each metabolite in the QC samples exceeded twice the mean concentration (two standard deviations for more than half of the metabolites), the subsequent sequence was reanalyzed. In total, 2547 QC samples for cations and 2627 samples for anions were used from Waves 1–3, for approximately 9 years from April 2012 to August 2021.

### 2.4. Variables Definition

Alcohol consumption and smoking status data were collected using self-administered questionnaires. Participants were asked to choose never, ex-, or current; never and ex- were defined as non-current and distinguished from current. Hypertension was defined as systolic blood pressure ≥ 140 mmHg, diastolic blood pressure ≥ 90 mmHg, or taking antihypertensive medication. Diabetes mellitus was defined as either fasting blood glucose ≥ 126 mg/dL, HbA1c (National Glycohemoglobin Standardization Program) ≥ 6.5%, or the use of hypoglycemic drugs or insulin. Dyslipidemia was defined as one or more of the following findings: low-density lipoprotein-cholesterol ≥ 140 mg/dL, triglyceride ≥ 150 mg/dL, high-density lipoprotein-cholesterol < 40 mg/dL, or the use of dyslipidemia drugs.

### 2.5. Statistical Analyses

For samples where metabolites were not detected, half of the lowest detected values were imputed [15]. To evaluate the reproducibility of data measurement, we calculated the coefficient of variation (*CV*) of the QC samples by dividing the variance by the mean. The intraclass correlation coefficient (*ICC*) was calculated to assess the reliability of metabolite profiling. Approximate *ICC*s were calculated as previously described [10].

$$Approximate\;ICC=1-\frac{{\left(CV_{QC}\right)}^{2}}{{\left(CV_{Participant}\right)}^{2}}\;$$

The rate of change in QC samples measured between Waves 1 and 2 and between Waves 1 and 3 was calculated as follows: (mean value of Wave 2 − mean value of Wave 1)/mean value of Wave 1, and (mean value of Wave 3 − mean value of Wave 1)/mean value of Wave 1.

Regarding the index of reliability, a *CV* > 30% is generally considered undesirable and a *CV* < 20% is considered desirable. Further, an *ICC* < 0.4 is considered poor, between 0.4 and 0.75 as fair to good, and above 0.75 as excellent [16]. In this study, three indices, the *CV* of QC, estimated *ICC*, and change rate of QC, were used to classify the reliability of each metabolite into the following three categories: excellent, fair to good, and poor. Specifically, reliability was considered as (i) excellent with *CV* < 20%, *ICC* ≥ 0.75, and change rate < 5%; (ii) fair to good with 20% ≤ *CV* < 30%, 0.4 ≤ *ICC* < 0.75, or 5% ≤ change rate < 10%; or (iii) poor with *CV* ≥ 30%, *ICC* < 0.4, or change rate ≥ 10%. The excellent and fair to good metabolites were comparable.

For metabolites classified as excellent and fair to good, we performed a linear mixed model stratified by sex and examined intra-individual changes in plasma metabolite concentrations per year at three time points over a 6-year period. The observed metabolite concentration was set as the dependent variable, years of follow-up as the independent variable, age at Wave 1 as the fixed effect, and individual ID as the variable effect. The subjects for analysis were those who participated in all three surveys, and a mixed model was constructed using all measured values from Waves 1 to 3. The estimates were divided by the standard deviation of Wave 1 for each metabolite. Additionally, we performed a sensitivity analysis on those who were free of diabetes from Wave 1 to the follow-up period. We calculated *p*-values using the Benjamini–Hochberg false discovery rate (FDR) method [17], a commonly used approach for testing multiple hypotheses [18]. Statistical significance was set at an FDR *p* < 0.05. All statistical analyses were performed using R.4.3.2 (R Foundation for Statistical Computing, Vienna, Austria).

## 3. Results

### 3.1. CV of QC Samples, ICC, and Change Rate of QC Samples

**Figure 2:**
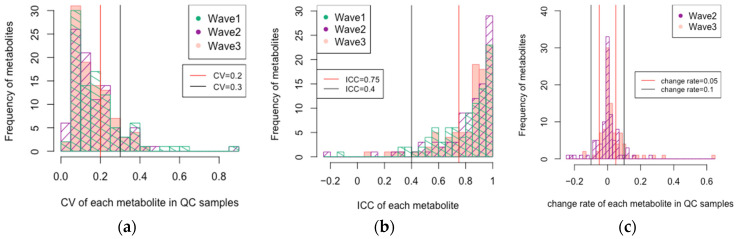
Histograms of the three indicators of reliability: (**a**) CV of QC; (**b**) approximate ICC; (**c**) change rate of QC. CV, coefficient of variation; ICC, intraclass correlation coefficient; QC; quality control samples.

The *CV*, *ICC*, and change rate of QC samples are shown in Figure 2 and Figure S1, and Table S1. The histograms indicated that the *CV*s were <0.3 (30%) for many metabolites (Figure 2). When the cations and anions were compared, the distribution of *CV*s for cations shifted to the left compared to that for anions, indicating smaller *CV*s for cations (Figure S1). A comparison of the percentage of metabolites with *CV* < 30% across the three surveys showed that 80 metabolites in Wave 1 (85%), 83 in Wave 2 (88%), and 86 in Wave 3 (91%) had *CV*s below 30%. Thus, the measurements became more stable as follow-up progressed. Regarding *ICC*, 87 metabolites (93%) were >0.4 in all three surveys, and 59 metabolites (63%) were >0.75. The change rates in QC between baseline and follow-up surveys were <10% for 78 metabolites (83%) and <5% for 54 metabolites (57%) at both follow-up visits. When the metabolites corresponding to the metabolism of amino acids and the other metabolites were compared, the median values of three indices were as follows: *CV*, 7.4% vs. 16.3%; *ICC*, 0.90 vs. 0.84; the change rates were 0.2% vs. 0.6%, respectively. This suggested that the metabolites related to the metabolism of amino acids were more stable.

Among the 94 metabolites, 39 (42%) were classified as excellent, 32 (34%) as fair to good, and 23 (24%) as poor. When stratified by cations and anions, 83% of the cations and 65% of the anions met the fair to good criteria or better.

### 3.2. Characteristics of the Study Participants and Sample Collection/Measurement Process

Characteristics of the participants from Wave 1 are presented in Table 1. The mean age ± standard deviation (SD) was 54.8 ± 10.5 years, and the mean body mass index ± SD was 23.0 ± 3.3 kg/m^2^. The proportion of current drinkers/smokers and the prevalence of lifestyle-related diseases were similar to the standard prevalence rates in Japan [19]; thus, the population was considered a general community-dwelling population. During the follow-up period, the distribution of body mass index in the study population remained unchanged (Wave 1: 23.0 ± 3.3 kg/m^2^, Wave 3: 23.4 ± 3.5 kg/m^2^), whereas the proportion of current drinkers and smokers decreased (drinkers, Wave 1: 51.5%, Wave 3: 50.2%; smokers, Wave 1: 16.9%, Wave 3: 13.4%), and the prevalence of lifestyle-related diseases increased (hypertension, Wave 1: 36.1%, Wave 3: 49.8%; diabetes mellitus, Wave 1: 6.9%, Wave 3: 10.1%; dyslipidemia, Wave 1: 45.6%, Wave 3: 52.3%).

### 3.3. Intra-Individual Changes in Metabolites over Time

**Figure 3:**
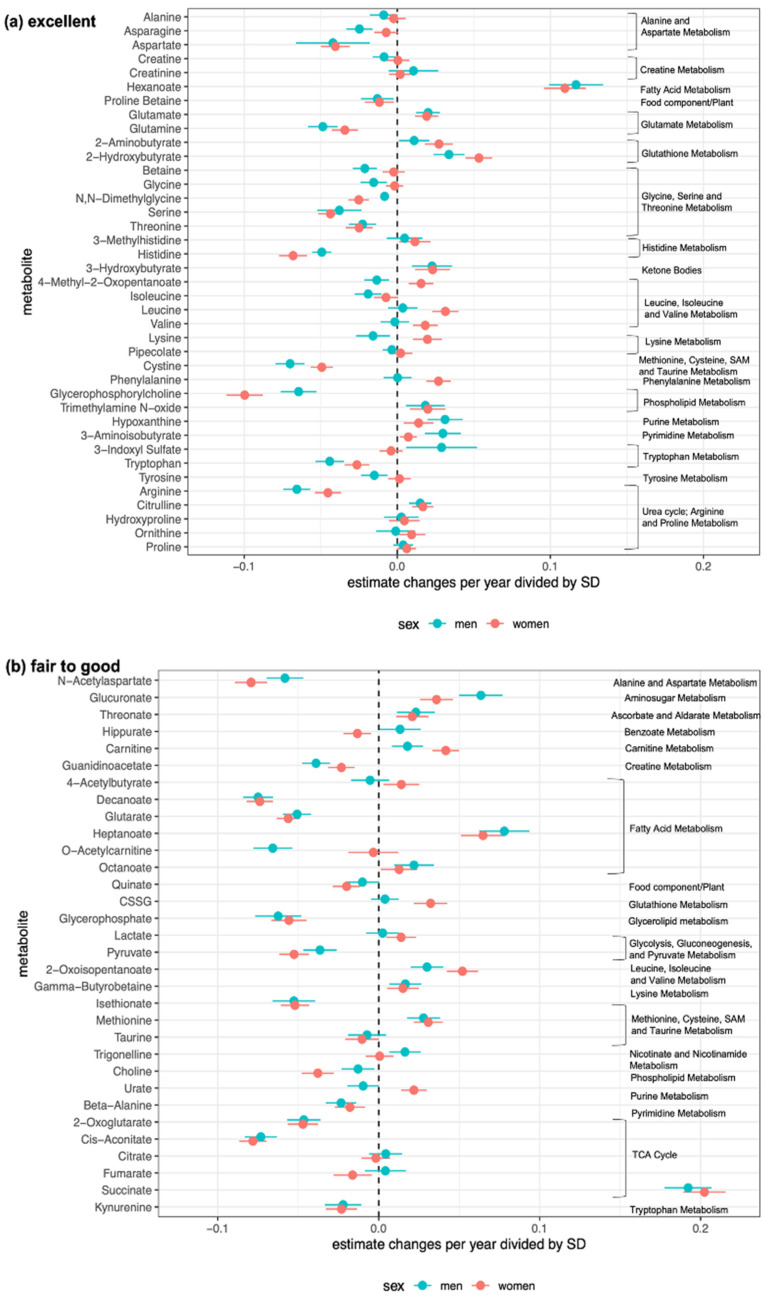
Intra-individual metabolite concentration changes per year at three time points over a 6-year period. Metabolites classified as having (**a**) “excellent” or (**b**) “fair to good” reliability for evaluation over time are illustrated. For each metabolite, point estimates of annual changes divided by standard deviation and their 95% confidence intervals are shown. The metabolic pathway is shown on the right side [20,21]. CSSG, cysteine-glutathione disulfide; SAM, S-Adenosylmethionine; SD, standard deviation; TCA, tricarboxylic acid cycle.

Figure 3 shows the results of the secular changes per year in 71 metabolites (76%) classified as having “fair to good” or “excellent” reliability over time. Point estimates of annual changes divided by standard deviation and their 95% confidence intervals are shown. The analysis was stratified according to sex. During the follow-up period, 20 and 32 metabolites significantly increased and decreased, respectively, in men, whereas 28 and 26 metabolites significantly increased and decreased, respectively, in women. More details are listed in Table S2, which is divided into cations and anions.

Of the 39 metabolites with “excellent” reliability over time, 29 metabolites (74.4%) in men and 26 metabolites (66.7%) in women exhibited significant changes during the follow-up period. Similarly, of the 32 “fair to good” metabolites, 23 (71.9%) in men and 28 (87.5%) in women showed significant changes. These results indicate that approximately 70% of the metabolites that were highly reliable in quantifying time-series concentrations showed clear changes over time. These results were consistent in a sensitivity analysis restricted to diabetes-free participants (Table S3).

## 4. Discussion

In our large-scale epidemiological study using a CE-MS metabolomics platform with excellent reproducibility, many metabolites demonstrated stable measurements of accurate absolute concentrations, even in longitudinal repeated measurements. This was made possible by using stable standards for all metabolites to determine absolute concentrations, strictly limiting the instruments used to reduce measurement errors, regularly checking instrument sensitivity, and reanalyzing samples when QC sample concentrations did not match the standards to maintain measurement quality. The *CV*s of 78 metabolites (83%) were less than 30% in all three surveys, which was comparable in reliability to the Wave 1 metabolome measurement data of 10,000 individuals previously reported [10].

Although attempts have been made to apply statistical corrections to measurement results when conducting large-scale profiling [7], statistically separating errors caused by the measurement process from true biological variation is more difficult, particularly in longitudinal repeated measurements. Therefore, the use of platforms for quantifying metabolite concentrations with high reproducibility is particularly important. The target of the present study was a single cohort established specifically for conducting metabolomic analysis; standardized sample collection and storage methods were used consistently throughout Waves 1, 2, and 3. Furthermore, by establishing standard operating procedures, limiting the equipment used, and monitoring measurements with QC samples, high reliability can be achieved even for longitudinal measurements.

*CV* and *ICC* have been used as criteria for reproducibility in conventional studies [8,22,23]. In the present study, we considered it important that the rate of change in QC samples be below a certain level in longitudinal studies; thus, the change rate of QC was added to the conventional criteria to better assess the reliability of metabolome data in longitudinal settings. A metabolite was defined as having excellent reliability if it met the criteria for all three indicators: *CV*, *ICC*, and change rate of QC. In total, 2% of the metabolites met the criteria for excellent reliability, and 34% met the criteria for fair to good reliability using all three indicators. Overall, 76% of all quantified metabolites could be evaluated for their multi-year changes in a longitudinal epidemiological study.

Consistent with previous cross-sectional studies, measurement reliability differed between cations and anions. In total, 83% of the cation metabolites met the “fair to good” or “excellent” criteria, compared to 65% of the anion metabolites. Furthermore, 63% of the cations met the “fair to good” criteria, compared to 12.5% of the anions. Overall, the plasma concentrations of anions were lower than those of cations, as reflected by the higher proportion of anions with concentrations below the limit of detection (4.7% for cations and 11.5% for anions). Smaller peaks hinder distinction of the peak areas from noise, making it challenging to quantify them with sufficient precision [6]. Studies using LC-MS have also shown that metabolites with large peak areas exhibit low *CV*s [23], and the reliability of measuring metabolites at low concentrations remains a topic for future research.

This study is valuable because it demonstrates the changes in metabolite concentrations over time with reliable measurements. Approximately 70% of the metabolites with excellent measurement reliability showed clear shifts over the 6-year time series, indicating that the changes were robust. Although much of the plasma metabolome is assumed to fluctuate over time, in this cohort we demonstrated the ability to detect epidemiological changes over time and the importance of focusing on these long-term changes.

Of the 71 metabolites classified as excellent or fair to good, 20 (28%) and 28 (39%) increased significantly in men and women, whereas 32 (45%) and 26 (37%) decreased significantly, respectively. Various metabolites belonging to specific pathways changed in the same direction in both men and women. Metabolites related to glutathione metabolism (2-aminobutyrate and 2-hydroxybutyrate) were significantly increased in both men and women, whereas metabolites related to alanine and aspartate metabolism (aspartate and N-acetylaspartate); glycine, serine, and threonine metabolism (serine, threonine, and N,N-dimethylglycine); and those related to tryptophan metabolism (tryptophan and kynurenine) were significantly decreased in both sexes. In contrast, 4-methyl-2-oxopentanoate, which is produced by the deamination of leucine, demonstrated different orientations in men and women, with a significant increase in women, but a decrease in men. Consistently, sex differences in leucine metabolism during exercise have been previously reported [24]. The present study showed that leucine levels were significantly increased in women during the follow-up period, but not in men.

This study has a few limitations. First, our metabolomics data were derived from a single cohort under a strict protocol. Not every cohort can follow the regulations established in TMCS, including those regarding sample collection and exclusive use of instruments. Thus, its feasibility in other prospective cohorts may remain a challenge. Nevertheless, the present study showed that if measurements were adequately performed under a strict protocol, the reliability of repeated metabolome measurements could be ensured over a period of as long as 6 years. Second, our data were limited to metabolites profiled solely using CE-MS. The measurement of other metabolites, such as lipids, was not examined. Future validations using other platforms, such as LC-MS and nuclear magnetic resonance, will be important to examine changes in a wider range of metabolites over time. Third, we demonstrated changes in metabolomic profiles over 6 years, adjusted only for age. This study aimed to examine the crude changes in metabolites in the general population without malignancy or cardiovascular disease at baseline (Wave 1). The results of the sensitivity analysis in the absence of diabetes throughout the follow-up period were consistent, suggesting that most of the results shown in Figure 3 may be independent of diabetes. However, given that lifestyle habits, such as drinking, smoking, and related health conditions, such as hypertension and dyslipidemia, could greatly affect the metabolome [25,26], we hope to expand our longitudinal metabolomics data in this cohort to study the combined effects of various lifestyle habits and health conditions on metabolome changes in the future.

## 5. Conclusions

In conclusion, this large-scale cohort study demonstrated that the CE-MS platform used in TMCS has high reliability for measuring plasma metabolites even in repeated measurements after 3 and 6 years in a large-scale cohort study. It also showed that a longitudinal assessment was adequate for a majority of metabolites. Furthermore, many highly reliable metabolites changed significantly over time, indicating that repeated longitudinal metabolome measurements are valuable for epidemiological use.

## Figures and Tables

**Table 1:** Characteristics of the study participants.

	All (n = 2999)	Men (n = 1317)	Women (n = 1682)
Age (years)	54.8 ± 10.5	55.1 ± 10.4	54.6 ± 10.6
Body mass index (kg/m^2^)	23.0 ± 3.3	23.8 ± 3.0	22.4 ± 3.4
Any current alcohol intake	1544 (51.5%)	1022 (77.7%)	522 (31.1%)
Current smoker	507 (16.9%)	425 (32.4%)	82 (4.9%)
Hypertension	1079 (36.1%)	547 (41.6%)	532 (31.7%)
Diabetes mellitus	206 (6.9%)	132 (10.1%)	74 (4.4%)
Dyslipidemia	1367 (45.6%)	676 (51.3%)	691 (41.1%)

## Data Availability

Raw data could not be made publicly available because the study participants did not consent to make their information freely accessible. Based on these findings, the Ethics Committee for Tsuruoka Metabolomics Cohort Study (which includes representatives of Tsuruoka citizens, the administration of Tsuruoka City, a lawyer, and expert advisers) strictly inhibits any public data sharing. However, the datasets generated and/or analyzed during the current study are available from the corresponding author upon reasonable request.

## Supplementary Materials

The following supporting information can be downloaded at https://www.mdpi.com/article/10.3390/metabo14010077/s1: Figure S1: Histograms of the CV of QC samples (A), approximate ICC (B), and change rate of QC samples (C) stratified by cations (1) and anions (2). CV, coefficient of variation. QC, quality control. ICC, intraclass correlation coefficient. Table S1: CV of QC samples, approximate ICC, and change rate of QC samples. CV, coefficient of variation. QC, quality control. ICC, intraclass correlation coefficient. CSSG, cysteine-glutathione disulfide. SAM, S-Adenosylmethionine. TCA, tricarboxylic acid cycle. The approximate ICC was calculated as follows: 1 − (CV of QC samples)^2^/(CV of participant samples)^2^. The change rate of QC samples was calculated as follows: (mean value of Wave 2 − mean value of Wave 1)/mean value of Wave 1 and (mean value of Wave 3 − mean value of Wave 1)/mean value of Wave 1. Table S2: Intra-individual changes in metabolites over time. Table S3: Intra-individual changes in metabolites over time restricted to diabetes-free participants.
