# Supplementary material for: Reliability of Time-Series Plasma Metabolome Data over 6 Years in a Large-Scale Cohort Study
Source: Metabolites. 2024 Jan 22;14(1):77. doi: 10.3390/metabo14010077 (PMC10819202; doi:10.3390/metabo14010077)

Figure S1. Histograms of the CV of QC samples (**A**), approximate ICC (**B**), and change rate of QC samples (**C**) stratified by cations (1) and anions (2). CV, coefficient of variation. QC, quality control. ICC, intraclass correlation coefficient.

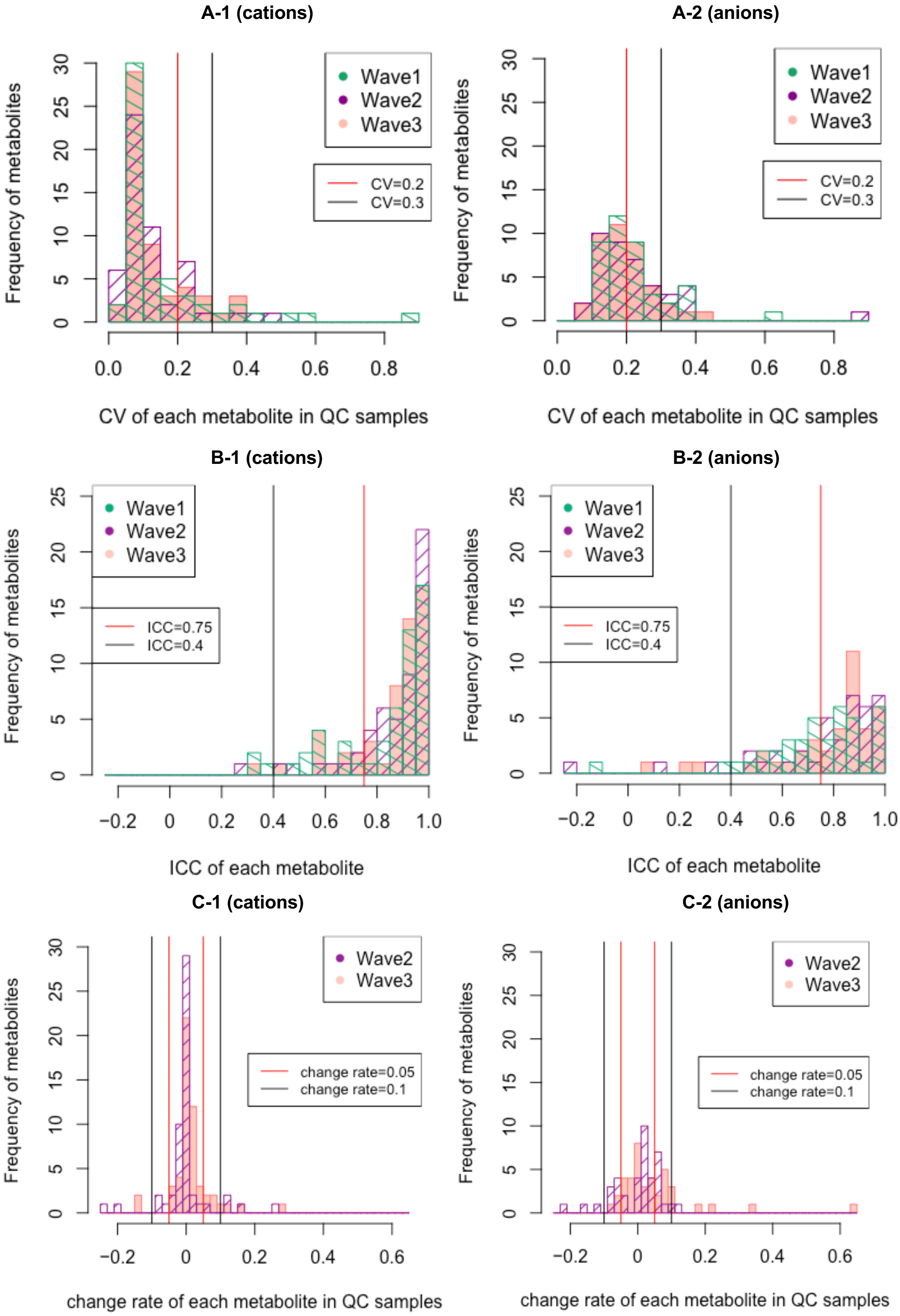

Supplement: Supplementary file 1 [file metabolites-14-00077-s001.zip › Supplementary file/Supplementary_Figure.pdf]
