# Supplementary figures and images for: Reliability of Time-Series Plasma Metabolome Data over 6 Years in a Large-Scale Cohort Study
Source: Metabolites. 2024 Jan 22;14(1):77. doi: 10.3390/metabo14010077 (PMC10819202; doi:10.3390/metabo14010077)

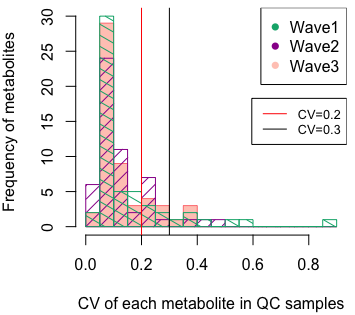

Supplement: Supplementary file 1 [file metabolites-14-00077-s001.zip › Supplementary file/Figure S1_a-1.tiff]

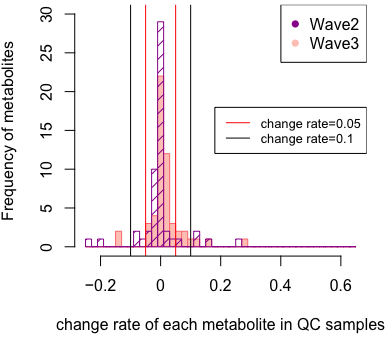

Supplement: Supplementary file 1 [file metabolites-14-00077-s001.zip › Supplementary file/Figure S1_c-1.tiff]

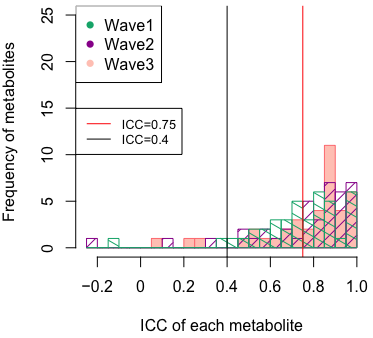

Supplement: Supplementary file 1 [file metabolites-14-00077-s001.zip › Supplementary file/Figure S1_b-2.tiff]

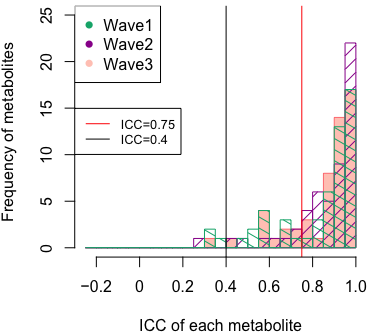

Supplement: Supplementary file 1 [file metabolites-14-00077-s001.zip › Supplementary file/Figure S1_b-1.tiff]

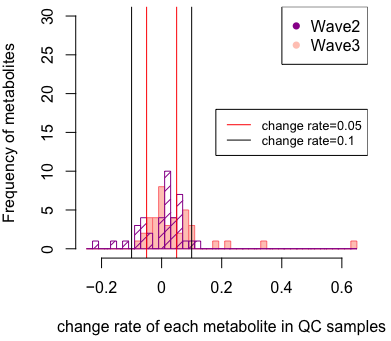

Supplement: Supplementary file 1 [file metabolites-14-00077-s001.zip › Supplementary file/Figure S1_c-2.tiff]

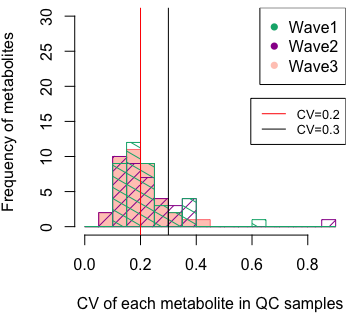

Supplement: Supplementary file 1 [file metabolites-14-00077-s001.zip › Supplementary file/Figure S1_a-2.tiff]
